# An Analysis of the Effect of *ABCA4* p.Asn1868Ile Genotypes on Retinal Structure in 26,558 Participants in the UK Biobank

**DOI:** 10.1167/iovs.64.7.31

**Published:** 2023-06-21

**Authors:** Mark J. Simcoe, Gavin Arno, Pirro G. Hysi, Tony Ko, Michel Michaelides, Christopher J. Hammond, Praveen J. Patel, Omar A. Mahroo, Andrew R. Webster, for the UK Biobank Eye and Vision Consortium

**Affiliations:** 1 Institute of Ophthalmology, University College London, London, United Kingdom; 2 NIHR Biomedical Research Centre at Moorfields Eye Hospital NHS Foundation Trust and the UCL Institute of Ophthalmology, London, United Kingdom; 3 Department of Ophthalmology, King's College London, St Thomas’ Hospital Campus, London, United Kingdom; 4 KCL Department of Twins Research and Genetic Epidemiology, London, United Kingdom; 5 Topcon Healthcare Solutions, Inc., Oakland, New Jersey, United States

**Keywords:** ABCA4, central retinal thickness, inherited retinal diseases

## Abstract

**Purpose:**

To determine whether the *ABCA4* retinopathy-associated variant p.Asn1868Ile (c.5603A>T) is associated with retinal structure or subclinical disease among the general population.

**Methods:**

UK Biobank participants of European ancestry with available spectral-domain optical coherence tomography (OCT) passing quality control metrics and exome sequencing data were included. Regression analyses using both linear and recessive models tested for the association between the p.Asn1868Ile variant and total retinal thickness, clinically relevant segmented layer thicknesses, and visual acuity. Further regression analyses were performed with automated quality control metrics to determine if the p.Asn1868Ile variant is associated with poor quality or abnormal scans.

**Results:**

Retinal layer segmentation and sequencing data for the p.Asn1868Ile variant were available for 26,558 participants, following exclusions. We identified no significant association between the p.Asn1868Ile variant and retinal thickness, any of the segmented layers, or visual acuity. There was also no significant difference for homozygous p.Asn1868Ile when tested under the assumption of a recessive model. No association was identified for any of the quality control metrics, and a χ^2^ test showed that participants with the p.Asn1868Ile variant were not more likely to be excluded during quality control due to poor quality scans (*P* = 0.56).

**Conclusions:**

The p.Asn1868Ile variant does not appear to affect the retinal structure or have pathogenic or subclinical effects on its own within the general population. The variant is likely to require other specific *cis*- or *trans*-acting modifying factors to cause ABCA4 retinopathy.

Biallelic pathogenic variants of the human *ABCA4* gene cause a spectrum of recessive inherited dystrophies, ranging from those limited to the central macula to others progressing to affect the entire retina and cause complete blindness.^1^^,^^2^ *ABCA4* variants cause more inherited disease than any other single gene in most cohorts.^3^

The *ABCA4* (ATP-binding cassette, subfamily A, member 4) gene is expressed in photoreceptors and encodes a protein involved in the clearance of all-*trans* retinaldehyde from photoreceptor discs into the cytoplasm for reduction to its alcohol.^4^^,^^5^

The *ABCA4* variant p.Asn1868Ile (c.5603A>T) has long been shown to be enriched in those with retinal disease compared to controls,^6^ but its pathogenicity is typically dependent on the co-occurrence of another cis-acting variant.^7^^,^^8^ The common allele frequency in European populations, which approaches 7%,^9^^,^^10^ precludes it acting by itself as a fully penetrant recessive allele. Work by Zernant et al.^11^, and others^7^^,^^8^ did show that the variant, when in trans with a distinct *ABCA4* variant explains a proportion of mildly affected patients. Furthermore, functional assays show the allele impairs ATP-driven transport of retinaldehyde conjugates.^5^

The purpose of this study is to determine whether the p.Asn1868Ile allele is associated with any subclinical pathogenic effects in the macula within the general population that do not have *ABCA4* retinopathy, using a large population-based cohort.

Participants from the UK Biobank data resource were used for this investigation. The UK Biobank is a volunteer cohort in the United Kingdom with genetic, medical, and lifestyle information for 503,325 participants aged between 40 and 69 years of age at recruitment.^12^ An enhanced ocular assessment including spectral-domain optical coherence tomography (OCT) imaging was performed on a subset of UK Biobank Eye and Vision Consortium participants.

## Methods

This is a cross-sectional analysis using the UK Biobank data resource.^12^

### Ethical Approval

All participants included in this analysis provided full informed consent in accordance with ethical approval granted and overseen by the UK Biobank Ethics and Governance Council. The UK Biobank study was conducted with the approval of the North-West Research Ethics Committee (ref 06/MRE08/65), in accordance with the principles of the Declaration of Helsinki. This research has been conducted using the UK Biobank Resource under Application Number 2112.^13^

### Participants

**Figure 1.**
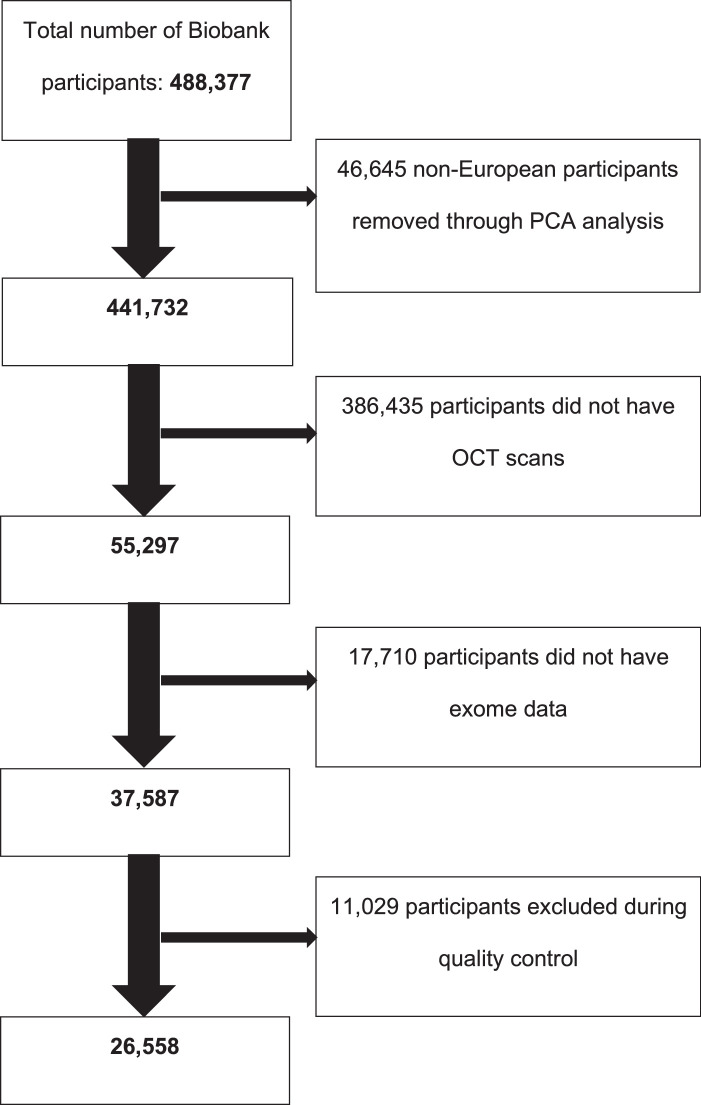
Flowchart of exclusions in forming the final cohort. PCA, principal component analysis.

All participants included in this analysis were confirmed to be of European ancestry using principal component analysis. All included subjects were part of the ∼200,000 interim, exome data release and the subset of participants who underwent macular spectral-domain OCT imaging. Prior to the application of exclusion criteria, a total of 37,586 participants were included for this analysis. The selection criteria for participants used in this study are outlined in Figure 1.

### Genotyping

Participants included in this analysis were genotyped using whole-exome sequencing performed by the UK Biobank Exome Sequencing Consortium. Sequencing used the GRCh38 assembly with an average of 20× coverage at 95.6% of sites.^14^ This included direct genotyping of the c.5603A>T, p.Asn1868Ile variant.

### Phenotyping and Exclusion Criteria

Macular spectral-domain OCT imaging was performed using the Topcon (Topcon GB, Newbury, Berkshire, UK) 3D OCT1000 Mark II. Imaging was performed in a dark room without pupil dilation using the three-dimensional 6 × 6-mm^2^ macular volume scan mode (512 A scans per B scan; 128 horizontal B scans in a raster pattern). The right eye was scanned first. Version 1.6.1.1 of the Topcon Advanced Boundary Segmentation (TABS) algorithm^15^ was used to segment the retinal layers and calculate the thickness of each layer across retinal fields or subfields in early treatment diabetic retinopathy study (ETDRS) subfields. Several segmentation indicators and quality metrics are calculated during this process, consisting of image quality score; inner limiting membrane (ILM) indicator, which indicates the minimum localized edge strength around the ILM boundary across the scan; valid count, which measures the degree of clipping in the OCT scan's z-axis; and the minimum motion correlation and maximum motion factor, which calculate the Pearson correlations and absolute differences between the nerve fiber layer and full retinal thickness from each set of consecutive B-scans, which identifies blinks, eye motion artifacts, and segmentation failures.^13^

The segmented layers used as phenotypes for this analysis are summarized in Table 1, and the scan quality metrics used as outcome phenotypes for this analysis are summarized in Supplementary Table S1. The mean value for both eyes was used as the final phenotype.

**Table 1.** Summary of All Phenotypes in the UK Biobank Cohort

Phenotype	Mean	SD
Age, y	56.9	8.0
Sex, female, %	52.2	NA
Total retinal layer	277.9	14.8
RNFL	28.4	4.8
GC-IPL	74.4	6.2
RPE	25.9	8.6
Photoreceptor layer	47.5	2.9
Visual acuity (logMAR)	0.008	0.165

Units for thickness measurements are in microns. GC-IPL, ganglion cell–inner plexiform layer; RNFL, retinal nerve fiber layer.

The best-corrected visual acuity was measured in both eyes using logMAR at a distance of 4 m, or 1 m if the participant was unable to read any letters at 4 m.^16^ The mean value of both eyes was used as the final phenotype for analysis.

Strict quality control criteria were applied for the OCT-derived measures, based on previously implemented methods.^13^ In brief: image quality score <45, worst 20% for ILM indicator, valid count, minimum motion correlation, maximum motion delta, and maximum motion factor. These strict cutoffs were selected to ensure poor-quality scans were excluded as any artifacts arising from low scan quality would reduce sensitivity of the analyses to detect subclinical effects. Additionally, participants were excluded if they reported one of the following ocular pathologies that may influence retinal thickness: high myopia (<−6 diopters), high hyperopia (>6 diopters), glaucoma, ocular hypertension, and AMD. Following these exclusion criteria, 26,558 participants were included in the final data set. A breakdown of the numbers excluded split by p.Asn1868Ile genotype is provided in Table 2.

**Table 2.** Summary of the Numbers of Participants in the Cohort Based on Genotype for the p.Asn1868Ile Variant for Which T is the Wild-Type Allele and A is the *ABCA4* Retinopathy-Associated Variant

Genotype	Pre-QC, *n*	Post-QC, *n*	% Removed
Homozygous T	32,629	23,026	29.4
Heterozygous	4770	3396	28.8
Homozygous A	187	136	27.3

“% removed” is the percentage of participants removed from each genotype grouping during quality control (QC).

### Statistical Analysis 

All statistical analyses were performed in R^17^ v3.6.3. Two models were used to test for association between p.Asn1868Ile genotype and ocular outcome (macula thickness, scan quality, and visual acuity).

The first was a linear model, corresponding to an additive effect at the locus, adjusted for age and sex, in which the ocular outcome was the outcome variable, and p.Asn1868Ile genotype was coded as the number (0, 1, 2) of minor alleles, c.5603A, present in the participant's genotype.

The second model was a recessive model, adjusted for age and sex, in which the ocular outcome was the outcome variable. However, in this model, p.Asn1868Ile genotype was coded as 0 for participants with either TT or TA genotypes, and participants with AA genotypes were coded as 1. This model identifies associated effects when allelic effects follow a recessive model of inheritance.

The following equation summarizes both models:

$$\begin{array}{c} \mathrm{Ocular}\;\mathrm{outcome}∼p.\mathrm{Asn}1868\mathrm{Ile}\;\mathrm{genotype}+\mathrm{age}+\mathrm{sex} \end{array}$$

Power calculations of the smallest effect sizes identifiable for each trait are provided in Supplementary Table S2.

## Results

The number of participants with each p.Asn1868Ile genotype is shown in Table 2. The p.Asn1868Ile genotypes within the UK Biobank (Table 2) are in Hardy–Weinberg equilibrium (χ^2^ = 0.792, *P* = 0.37) indicating that this cohort is unlikely subject to strong sampling bias for the purpose of this study.

Participants carrying the p.Asn1868Ile allele were not more likely to be excluded due to poor-quality scans than those without (Table 2, χ^2^ = 1.17, *P* = 0.56). Therefore, all results reported will be derived from the data set after the application of exclusion criteria.

**Figure 2.**
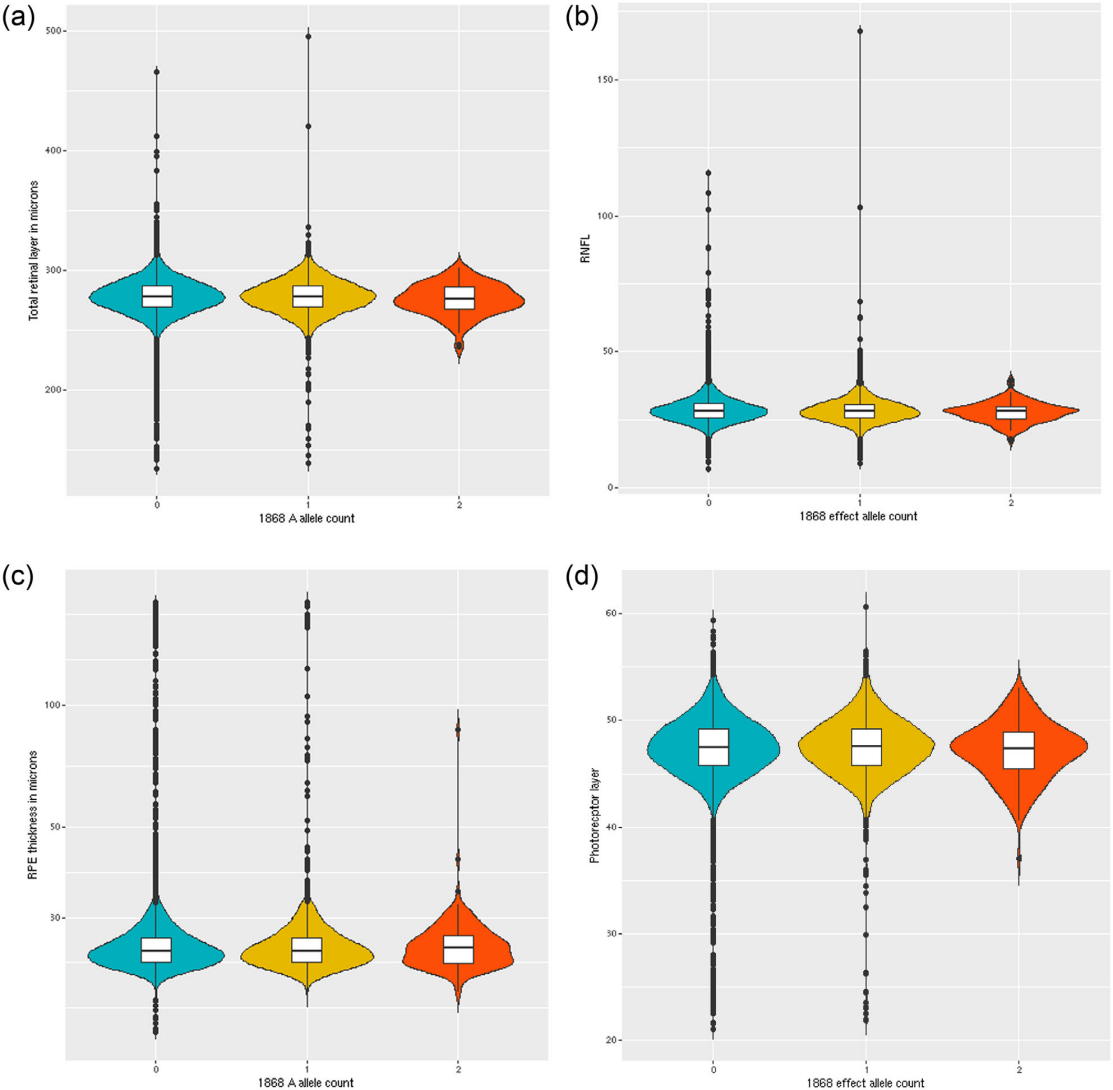
The “A” allele is referring to the allele at the genotype. (**a**) Violin plot of association results between p.Asn1868Ile and total retinal thickness. (**b**) Violin plot of association results between p.Asn1868Ile and retinal nerve fiber layer. (**c**) Violin plot of association results between p.Asn1868Ile and retinal pigment epithelium. **(d)** Violin plot of association results between p.Asn1868Ile and the photoreceptor layer. For all panels, the *white box* in the center of each violin shows the interquartile range and the *central line* in the *white box* is the median. The colored region around this box is a density plot capturing 99.3% of values.

The linear model identified no significant association between the total retinal thickness and p.Asn1868Ile allele prior to corrections for multiple testing (β = −0.12, SE = 0.25, *P* = 0.62) (^1^Fig. 2a). There was no evidence of association for specific segmentations of the retina, including the retinal nerve fiber layer (β = −0.12, SE = 0.79, *P* = 0.13) (Fig. 2b), ganglion cell–inner plexiform layer (β = −4.4 × 10^−3^, SE = 0.10, *P* = 0.97), and, most important, the RPE (β = 0.097, SE = 0.15, *P* = 0.51) (Fig. 2c) and the photoreceptor layer (β = −0.017, SE = 0.049, *P* = 0.72) (Fig. 2d), which are the retinal layers most affected in *ABCA4* retinopathy. All linear association results are summarized in Table 3.

**Table 3.** Summary of Linear Regression Results for Thickness Measurements and Visual Acuity

Phenotype	Beta	SD	*P*
Total retinal layer	−0.124	0.250	0.62
RNFL	−0.121	0.079	0.13
GC-IPL	−0.004	0.104	0.97
RPE	0.097	0.146	0.51
Photoreceptor layer	−0.017	0.049	0.724
Visual acuity (logMAR)	−5.99E-04	0.003	0.83

We tested for an association using a linear model between p.Asn1868Ile and all eight OCT quality control (QC) measures, which might serve as a proxy for structural abnormalities in the retina. Prior to corrections for multiple testing, no significant association was identified for image quality (β = −0.048, SE = 0.15, *P* = 0.76) (Supplementary Fig. S1) or any of the other seven QC metrics (Supplementary Table S3).

The final test using the linear model did not identify any association between p.Asn1868Ile and visual acuity (β = −6.0 × 10^−4^, SE = 3.0 × 10^−3^, *P* = 0.83).

Finally, we repeated all association tests using the recessive model. No association was identified between all retinal thickness measurements or visual acuity under the assumption of recessive effects (all summary statistics provided in Table 4). Tests for the association between all OCT quality control measures and p.Asn1868Ile also produced negative results (Supplementary Table S4).

**Table 4.** Summary of Recessive Model Results for Thickness Measurements and Visual Acuity

Phenotype	Beta	SD	*P*
Total retinal layer	−1.100	0.626	0.08
RNFL	−0.340	0.195	0.08
GC-IPL	−0.236	0.260	0.37
RPE	0.074	0.367	0.84
Photoreceptor layer	−0.178	0.122	0.15
Visual acuity (logMAR)	−1.15E-03	6.94E-03	0.87

Interestingly, of the 187 participants homozygous for the c.5603A allele (prior to exclusion), only 2 participants (1%) were labeled with an *International Classification of Diseases, 10th Revision* (*ICD-10*) code suggesting retinal disease (*ICD-10* code H35.3: degeneration of macula and posterior pole). One of these two participants also had the p.Gly863Ala (c.2588G>C) allele, which has been reported as pathogenic in *cis* with p.Asn1868Ile.^7^

## Discussion

In this study, we provide evidence that the *ABCA4* retinopathy-associated missense allele, p.Asn1868Ile, is not associated with retinal structure (as quantified by macular layer thicknesses) or central retinal function (as quantified by visual acuity) within the general population, under the assumptions of both an additive and a recessive model.

Across many diseases, mild variants are often associated with subclinical effects in endophenotypes for the disease in carriers. Negative results in this analysis are important as they show that p.Asn1868Ile pathogenicity functions entirely through interactive effects and not through additive effects.

Negative association results do not necessarily mean that the two are not associated; it may result from insufficient statistical power. However, given our large cohort of 26,558 participants, this study was sufficiently powered to identify small effects (Supplementary Table S2); therefore, these negative results provide strong evidence that the p.Asn1868Ile allele is not associated with subclinical retinal effects among the general population. The absence of significance without applying corrections for multiple testing further strengthens this conclusion.

*ABCA4* retinopathy is a rare disease; given the size of our cohort, we would expect a small number of cases to be present in our sample. *ICD-10* code data indicated that 2 of the 187 p.Asn1868Ile homozygotes may have *ABCA4* retinopathy; however, a more thorough and comprehensive assessment of OCT scans is required to accurately determine the specific macular condition these cases have. The additional information required for this was not available. All other participants with this genotype had no record of retinal disease that could be *ABCA4* retinopathy. This supports that this cohort is appropriate for investigating whether this allele is associated with retinal structure in the general population.

It has been hypothesized that *ABCA4* variants may contribute to AMD risk,^18^ with some evidence for the p.Asn1868Ile variant being associated with AMD risk.^19^^,^^20^ It is also well documented that AMD is strongly associated with retinal thickness, specifically within the RPE, macula, and photoreceptor layers.^21^^–^^24^ The lack of association between p.Asn1868Ile and retinal thickness for these layers suggests that it may not be an AMD risk factor, and this is consistent with AMD genome-wide association analyses that do not identify any association at *ABCA4.*^25^^,^^26^ It is important to consider that the studies supporting an association between p.Asn1868Ile were conducted with small sample sizes (212 and 54 participants, respectively),^19^^,^^20^ whereas this study and the AMD genome-wide association analysis (GWAS) analyses had sample sizes in the tens of thousands. The adoption of autofluorescence imaging in diagnosis since a link between AMD and *ABCA4* was first hypothesized has also shown that the hypothesized link is a consequence of disease misclassification, as autofluorescence imaging allows for better discrimination between the two conditions.^27^^,^^28^

One limitation of the UK Biobank cohort is that it is not fully population representative, and participants included in this analysis are all ancestral Europeans aged 40 to 70. *ABCA4* retinopathy typically has an age of onset <20 years,^29^ much younger than the age of participants in our cohort. However, late-onset *ABCA4* retinopathy is diagnosed after the age of 45. Therefore, while we can be confident that the majority of our cohort will be correctly categorized as disease free, it is possible a small number may develop this condition in the future. Also, any potential selection bias in this cohort (e.g., in terms of those individuals who agree to participate having better or worse visual function) could apply to the present study.

In conclusion, the c.5603A>T, p.Asn1868Ile variant alone does not appear to affect total retinal thickness or exert any subclinical effects in the general population. The variant therefore requires a distinct pathogenic *cis-* or *trans*-allele to cause any pathogenic effects, not just retinopathy as previously described in patient studies.^7^^,^^11^

## Supplementary Material

Supplement 1

Supplement 2

Supplement 3
