# Supplementary material for: An Analysis of the Effect of ABCA4 p.Asn1868Ile Genotypes on Retinal Structure in 26,558 Participants in the UK Biobank
Source: Invest Ophthalmol Vis Sci. 2023 Jun 21;64(7):31. doi: 10.1167/iovs.64.7.31 (PMC10291893; doi:10.1167/iovs.64.7.31)
Supplement: Supplement 2 [file iovs-64-7-31_s002.pdf]

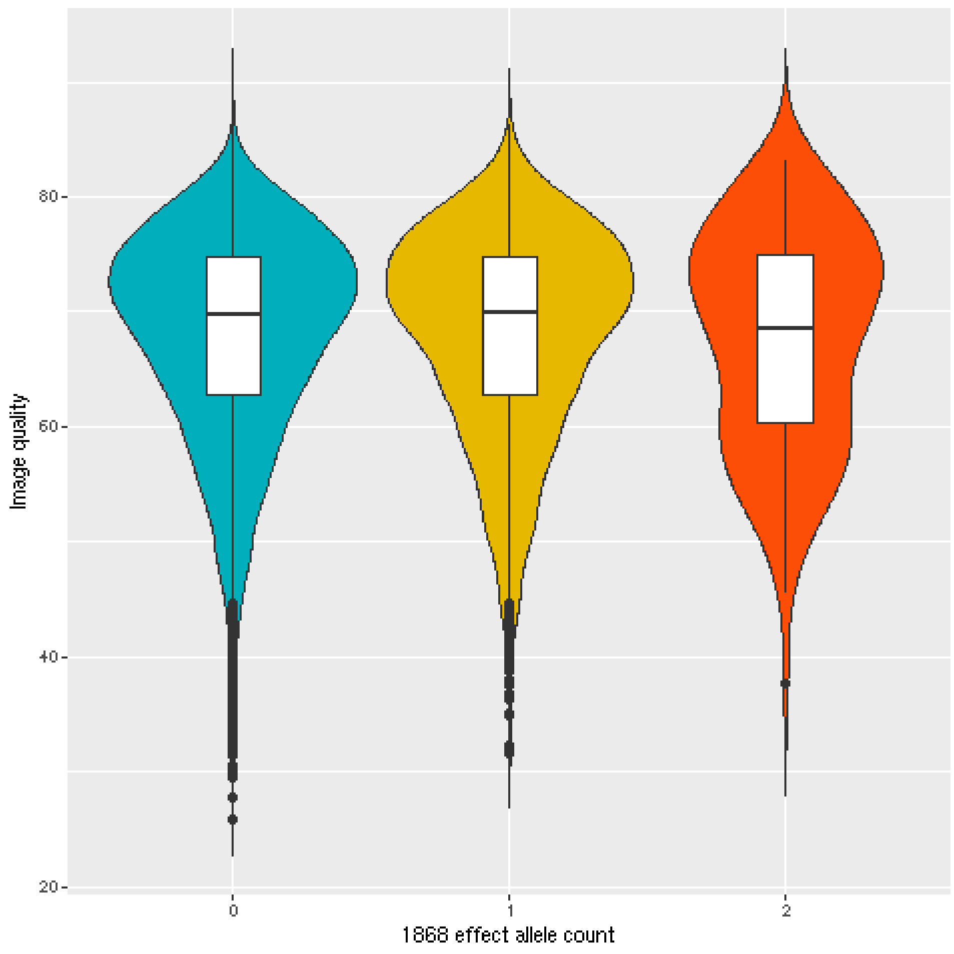

Supplementary Figure S1 – Violin plot of association results between p.Asn1868Ile and the image quality. The white box in the centre of each violin shows the interquartile range and the central line in the white box is the median. The coloured region around this box is a density plot capturing 99.3% of values.
